# A genetic screen for replication initiation defective (*rid*) mutants in *Schizosaccharomyces pombe*

**DOI:** 10.1186/1747-1028-5-20

**Published:** 2010-08-27

**Authors:** Alexandra M Locovei, Ling Yin, Gennaro D'Urso

**Affiliations:** 1 Department of Molecular and Cellular Phamacology, University of Miami School of Medicine PO Box 016189, Miami, FL 33140, USA

## Abstract

In fission yeast the intra-S phase and DNA damage checkpoints are activated in response to inhibition of DNA replication or DNA damage, respectively. The intra-S phase checkpoint responds to stalled replication forks leading to the activation of the Cds1 kinase that both delays cell cycle progression and stabilizes DNA replication forks. The DNA damage checkpoint, that operates during the G2 phase of the cell cycle delays mitotic progression through activation of the checkpoint kinase, Chk1. Delay of the cell cycle is believed to be essential to allow time for either replication restart (in S phase) or DNA damage repair (in G2). Previously, our laboratory showed that fission yeast cells deleted for the N-terminal half of DNA polymerase ε (Cdc20) are delayed in S phase, but surprisingly require Chk1 rather than Cds1 to maintain cell viability. Several additional DNA replication mutants were then tested for their dependency on Chk1 or Cds1 when grown under semi-permissive temperatures. We discovered that mutants defective in DNA replication initiation are sensitive only to loss of Chk1, whilst mutations that inhibit DNA replication elongation are sensitive to loss of both Cds1 and Chk1. To confirm that the Chk1-sensitive, Cds1-insensitive phenotype (*rid *phenotype) is specific to mutants defective in DNA replication initiation, we completed a genetic screen for cell cycle mutants that require Chk1, but not Cds1 to maintain cell viability when grown at semi-permissive temperatures. Our screen identified two mutants, *rid1-1 *and *rid2-1*, that are defective in Orc1 and Mcm4, respectively. Both mutants show defects in DNA replication initiation consistent with our hypothesis that the *rid *phenotype is replication initiation specific. In the case of Mcm4, the mutation has been mapped to a highly conserved region of the protein that appears to be required for DNA replication initiation, but not elongation. Therefore, we conclude that the cellular response to inhibition of DNA replication initiation is distinct from blocking DNA replication elongation, and this difference can be exploited to identify mutants specifically defective in DNA replication initiation.

## Background

Assembly of DNA replication complexes begins in early G1 following degradation of cyclin-dependent kinases at the conclusion of mitosis [1]. The dramatic drop in CDK activity following anaphase promotes the binding of Mcm2-7 to origin DNA to form pre-replicative complexes or pre-RCs. Formation of the pre-RC requires both Cdc18/Cdc6 and Cdt1 proteins [2-4]. Once the pre-RC is assembled, activation of CDK (cyclin dependent kinase) and DDK (Dbf4-dependent kinase) at the beginning of S phase leads to the binding of additional replication proteins to origin DNA including DNA polymerase epsilon, Sld2, Sld3, Cdc45, and GINS forming the pre-initiation complex or pre-IC [5,6]. Binding of DNA polymerase alpha is then required to allow synthesis of short primers that are then extended during S phase by DNA polymerase delta [7-10].

DDK and CDK are two serine kinases required for the onset of S phase. They target components of the pre-RC and pre-IC, respectively and are essential for initiation of DNA replication. It is believed that structural modifications to the pre-RC stimulated by DDK activity leads to Cdc45 binding to the MCM complex stimulating its helicase activity [11]. Following the partial unwinding of DNA, CDK then targets two proteins, Sld2 and Sld3, promoting the assembly of the pre-IC and ultimately the onset of S phase [12,13]. The Mcm2-7 complex is unique in that it appears to function both in the initiation of DNA replication ie. during formation of the pre-RC, as well as in DNA replication elongation as the putative DNA helicase [11,14]. These two functions appear to be separable, since Mcm mutants defective in DNA replication elongation are still capable of binding chromatin and presumably establishing the pre-RC [15]. Mcm proteins contain ATP binding sites but ATP hydrolysis is only required for replication elongation and not for chromatin binding [15].

When DNA replication is blocked by either addition of inhibitors such as hydroxyurea or by DNA damage, a checkpoint control ensures that mitotic entry is delayed and that replication complexes are stabilized [16,17]. In fission yeast, this checkpoint, called the intra-S phase checkpoint requires several proteins encoded by the *rad*, *hus1*, *mrc1 *and *cds1 *genes. Although Cds1 delays cell cycle progression by phosphorylating and inhibiting the Cdc25 phosphatase [18,19], its most critical role for maintaining cell viability is to stabilize stalled replication complexes [20-22]. In the absence of Cds1, cells lose viability rapidly upon inhibition of DNA replication elongation, and this decrease in viability is not the result of cells entering mitosis prematurely. On the other hand, DNA damage during G2 leads to the activation of a distinct checkpoint that is also dependent on the expression of the *rad *and *hus1 *genes, but requires a unique adaptor protein Crb2, and the effector kinase Chk1 [23,24]. Chk1 maintains cell viability by preventing premature entry into mitosis by directly phosphorylating and inhibiting Cdc25 [24].

Previously, our lab reported that cells deleted for the N-terminal half of DNA polymerase ε catalytic subunit (Cdc20) are viable, but have a significant S phase delay [25]. Although we expected that this delay would be due to activation of the intra-S phase checkpoint, we were surprised to find that these cells require Chk1, but not Cds1 to maintain cell viability. Our hypothesis was that the Chk1 dependency, Cds1-independency of the DNA polymerase epsilon mutant reflected a unique role for this polymerase in the assembly of the pre-IC. We therefore tested several other DNA replication temperature-sensitive (*ts*) mutants for their sensitivity to the loss of either Cds1 or Chk1 when grown under semi-permissive conditions. Our results suggest that mutants defective in DNA replication initiation (referred to as *rid *mutants) require the checkpoint kinase Chk1 for viability, while those defective in the elongation step (referred to as *red *mutants) require Cds1 [26]. To test our hypothesis further we have now completed a genetic screen for cell division cycle (*cdc*) mutants that are sensitive to the loss of Chk1, but not Cds1. This screen identified novel mutant alleles of *cdc30/orp1 *and *cdc21/mcm4*, two proteins that are essential for DNA replication initiation. Consistent with the screen being specific for initiation mutants we find that the mutation identified in Mcm4, which maps to a highly conserved region within the central MCM domain signature motif III (http://srs.ebi.ac.uk/srsbin/cgi-bin/wgetz?-e+[prints-AccNumber:PR01657), disrupts only its initiation function, whilst leaving its putative elongation function intact. Our results demonstrate that the *rid *mutant phenotype provides a powerful tool to identify mutants defective in the DNA replication initiation, and that the cellular response to inhibition of DNA replication initiation is distinct from the response to blocking DNA replication elongation.

## Results

### Replication initiation (*rid*) mutants have unique checkpoint requirements

Previously, we showed that several mutants defective in DNA replication initiation are sensitive to the loss of *chk1*^*+*^, but not *cds1*^*+ *^[26], see also Additional file 1, Table S1. In contrast, the viability of DNA replication elongation mutants was severely compromised in the absence of either Cds1 or Chk1 when grown at semi-permissive temperatures. We hypothesized that the Chk1-dependent, Cds1-independent phenotype is unique to replication initiation mutants and could be exploited in a genetic screen to identify mutants specifically defective in DNA replication initiation.

### Developing a screen for *rid *mutants

**Figure 1:**
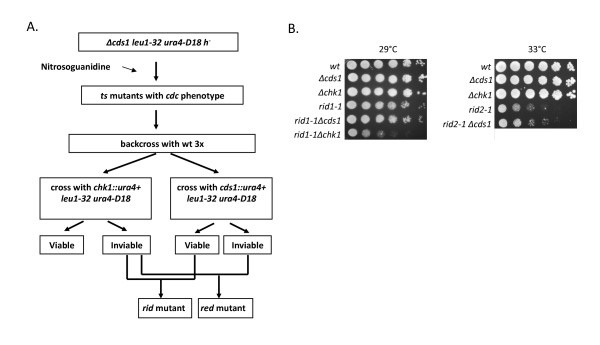
**Isolation of *rid *mutants**. (A) Strategy for identification or replication initiation defective (*rid*) mutants, see methods for details. (B) Left panel, serial dilution of *rid1-1 *at the semi-permissive temperature of 29°C, Right panel, serial dilution of *rid2-1 *at the semi-permissive temperature of 33°C. The mutant *rid2-1*Δ*chk1 *is not shown because it was found to be non-viable at 25°C.

The strategy for our genetic screen to identify DNA replication initiation mutants is outlined in Figure 1A. First, we randomly mutagenized cells with nitrosoguanidine and screened for mutants that displayed a cell cycle delay (indicated by an elongated cell phenotype) when shifted to the restrictive temperature of 36°C. In our initial screen, the Δ*cds1 *strain was used for mutagenesis to avoid isolating mutants that were defective in DNA replication elongation and thus sensitive to loss of Cds1. Selected mutants that displayed the *cdc *(*c*ell *d*ivision *c*ycle) phenotype were backcrossed to wild-type cells several times to eliminate the background Δcds1 and confirm that the mutation had occurred in a single gene. Each putative *rid *mutant was then crossed to either the Δ*cds1 *or Δ*chk1 *strain to generate double mutants. Using a serial dilution spot assay, the viability of the double mutants was then compared to both wildtype cells (wt) and the three parental single mutant strains (putative *rid *mutant, Δ*chk1 *and Δ*cds1*) at several different temperatures. Mutants that showed a selective loss of viability in the absence of Chk1, but not Cds1 as the temperature was increased were selected as potential *rid *mutants (Figure 1B). From approximately 70,000 colonies screened, 500 temperature sensitive mutants were isolated, of which 56 displayed the *cdc *phenotype upon temperature shift. Only two mutants, *rid1-1 *and *rid2-1*, displayed the *rid *phenotype ie. *cdc *phenotype and loss of viability in the absence of Chk1 while retaining viability in the absence of Cds1 as shown in Figure 1B. In the case of *rid2-1*, we were unable to isolate viable *rid2-1*Δ*chk1 *double mutants at 25°C suggesting that *rid2-1 *is defective in DNA replication initiation even at this lower temperature. In the absence of *chk1*, *rid2-1 *mutants are only capable of forming small colonies of 4-8 cells, and many of these cells display the *cut *phenotype, indicating that cells have entered mitosis in the absence of a complete round of DNA synthesis [27].

### Identification of *rid1-1 *and *rid2-1 *as novel alleles of *cdc30/orc1 *and *cdc21/mcm4*

**Figure 2:**
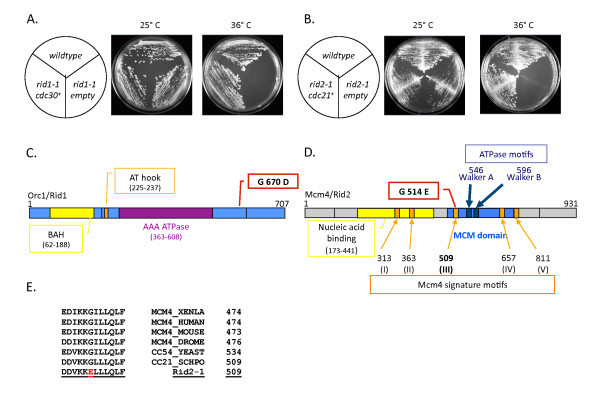
**Complementation of *rid *mutants with cloned DNA fragments**. (A) Wildtype or *rid1-1 *cells were transformed with plasmid DNA containing the *orc1*^*+ *^gene or no *orc1*^*+ *^(empty). Only those cells transformed with *orc1*^*+ *^containing DNA were capable of growing at the restrictive temperature of 36°C. (B) Wildtype or *rid2-1 *cells were transformed with plasmid DNA containing *mcm4*^*+ *^or no *mcm4*^*+ *^(empty). Only cells transformed with *mcm4*^*+ *^DNA were capable of growing at 36°C. (C) Schematic diagram of *rid1-1 *showing the position of the mutation in the C-terminal portion of Orc1. (D) Schematic diagram of *rid2-1 *showing the position of the mutation in Mcm4 signature motif III, relative to the Walker A and Walker B binding motifs. The number 509 corresponds to the first amino acid position for motif III. The Mcm4 signature motifs are 10-12 amino acids long. (E) Sequence comparison of motif III from various organisms including human indicating strong evolutionary conservation for this region of the protein. The position of the *rid2-1 *mutation is shown.

To identify the genes mutated in both *rid1-1 *and *rid2-1 *we transformed both mutants with a variety of plasmids expressing genes known to be essential for DNA replication. A plasmid expressing the *cdc30/orc1+ *gene rescued the *ts cdc *phenotype of *rid1-1 *(Figure 2A), and a plasmid expressing *cdc21*^*+*^*/mcm4*^*+ *^rescued *rid2-1 *(Figure 2B). Integration of the gene expressing *orc1*^*+ *^(in the *rid1-1 *strain) and *mcm4*^*+ *^(in the *rid2-1 *strain) showed that the gene was closely linked to the mutation, suggesting that *rid1-1 *and *rid2-1 *encode *orc1*^*+ *^and *mcm4*^*+ *^respectively (data not shown). To confirm the results of our complementation analysis, we isolated genomic DNA from *rid1-1 *and *rid2-1*, and cloned and sequenced the *cdc30 *and *mcm4 *genes, respectively. For *rid1-1 *we identified a single point mutation, G2061A that corresponds to an amino acid change from glycine to aspartic acid at position 670 in the Orc1 protein (Figure 2C). For *rid2-1*, we identified a point mutation, G1541A, corresponding to a change from glycine to glutamate at position 514 in Mcm4 (Figure 2D). The *rid1-1 *mutation is located at the C-terminus of the Orc1 protein, outside of any functional domain previously reported for this protein and downstream of the AAA ATPase domain. The mutation identified in Mcm4 resides in the central MCM domain, within the Mcm4 signature motif III, a highly conserved region of the protein (Figure 2E). No specific function has yet been ascribed to this domain. This mutation is adjacent to but not within the ATP binding Walker A motif that has been suspected of being required for MCM-associated helicase activity [28,29].

### The phenotype of the two *rid *mutants suggests defects in DNA synthesis

**Figure 3:**
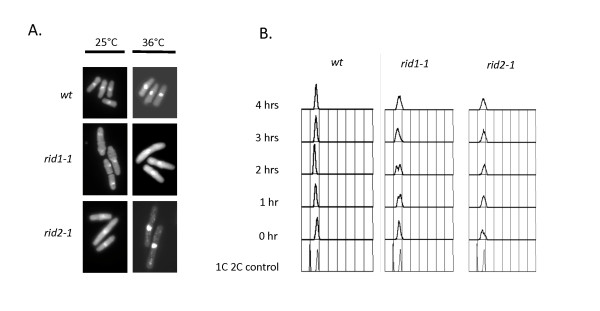
**Phenotype of *rid *mutants at restrictive temperature**. (A) *rid1-1 *and *rid2-1 *show slight elongation when shifted to the semi-permissive temperature, suggesting a cell cycle delay. (B) Flow cytometry analysis of *rid1-1 *and *rid2-1 *following shift to restrictive temperature of 36°C. Cells accumulate with 1C DNA content following shift of *rid1-1 *to 36°C (150-180 mins post-shift) whilst *rid2-1 *shows very little change in DNA content.

Both mutants display a *cdc *phenotype and eventually arrest with a single nucleus when grown at the non-permissive temperature of 36°C (Figure 3A). For *rid2-1*, even at the permissive temperature of 25°C, the cells are elongated when compared to wild-type cells consistent with our observation that Chk1 is required for viability even at this lower temperature. Upon shift to the restrictive temperature, analysis of DNA content by flow cytometry demonstrates that *rid1-1 *displays a shift towards 1C DNA content during the first two hours showing significant accumulation of cell is S phase at longer times. The mutant *rid2-1 *displays a broad peak at 25°C suggesting that cells are already delayed in S phase at the permissive temperature consistent with the observed elongated phenotype and sensitivity to loss of Chk1 at this temperature. Therefore, although both mutants are likely to have problems completing S phase, we conclude that neither of these mutants display a robust defect in replication initiation. We suspect that the observed S phase delay and activation of Chk1 are caused by either DNA damage that arises from collisions of replication forks with non-fired origins or from mis-assembly of replication initiation complexes. We hypothesize that this partial block to initiation that results from a failure to assemble the proper number of initiation complexes can ultimately inhibit fork progression and generate DNA damage related structures that can activate Chk1. It is important to point out that this scenario is presumed to be distinct from the Cds1-dependent checkpoint that responds to more extensive inhibition of DNA replication elongation as is observed in mutants defective in DNA polymerase delta (*cdc6*, *cdc27*) or in cells grown in the presence of hydroxyurea.

### Chk1 activation in *rid1-1 *and *rid2-1 *mutants

**Figure 4:**
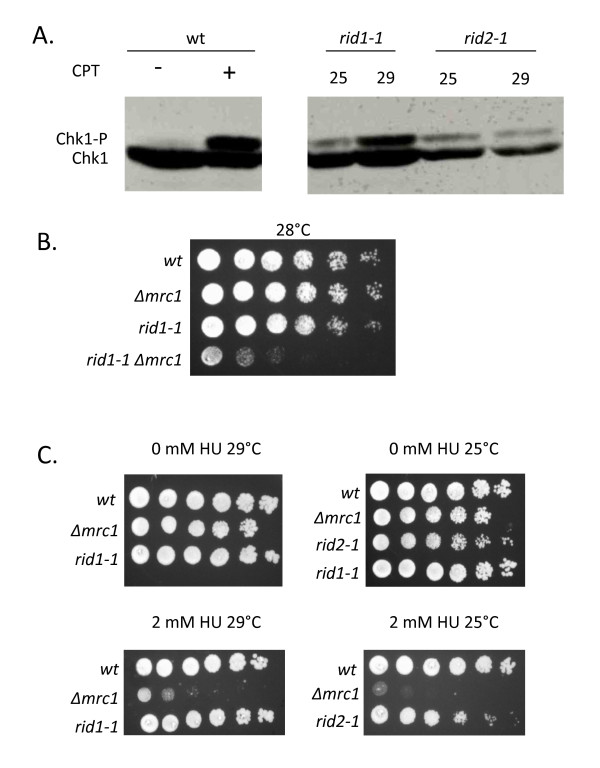
**Chk1 is activated in *rid1-1 *and *rid2-1***. (A) Chk1 phosphorylation occurs in both *rid1-1 *and *rid2-1 *strains at 29°C and 25°C, respectively. (B) *rid1-1 *viability is dependent on expression of *mrc1*^*+ *^(C) *rid1-1 *and *rid2-1 *are not hypersensitive to hydroxyurea treatment as compared to Δ*mrc1*.

Previous experiments in our lab showed that in addition to Chk1 being required for the viability of *rid *mutants grown at elevated temperatures, *chk1 *was also activated in these mutants [26]. Therefore we monitored Chk1 activation in the *rid1-1 *and *rid2-1 *mutants by assaying for the presence of the slower migrating phosphorylated form of Chk1 by western immuno-blotting at elevated temperatures. For these experiments cells containing HA-tagged Chk1 were crossed to each of the two mutant strains to generate *rid1-1 *and *rid2-1 *strains containing HA-Chk1 in place of wild-type Chk1. Exponentially growing cultures of these two mutant strains were shifted to their respective semi-permissive temperature for two hours, and cells were then harvested, protein extracts prepared and analyzed for Chk1 protein by western blotting using HA antibodies (Figure 4A). In both *rid1-1 *and *rid2-1*, the appearance of the Chk1-phosphorylated protein confirms that Chk1 is activated in these mutants at the elevated temperature. Not surprisingly, Chk1 is also phosphorylated in *rid2-1 *at 25°C, consistent with its sensitivity to the loss of Chk1 at this temperature.

### Mrc1 is required in *rid1-1 *and *rid2-1 *mutants

Our analysis of other *rid *mutants has demonstrated that their viability is not only dependent on Chk1, but also requires Mrc1 [30-34]. These experiments revealed a function for Mrc1 that is independent of its role in activation of the intra-S phase checkpoint. We confirmed that the viability of *rid1-1 *and *rid2-1 *strains is also dependent on expression of Mrc1 (Figure 4B). As was observed for Δ*chk1*, no viable double mutants containing *rid2-1 *and deletion of *mrc1 *could be obtained at 25°C. Consistent with our previous findings regarding initiation mutants, we also found that *rid1-1 *or *rid2-1 *were not hypersensitive to hydroxyurea (Figure 4C).

### The mutant *rid2-1 *is defective in DNA replication initiation, but not elongation

**Figure 5:**
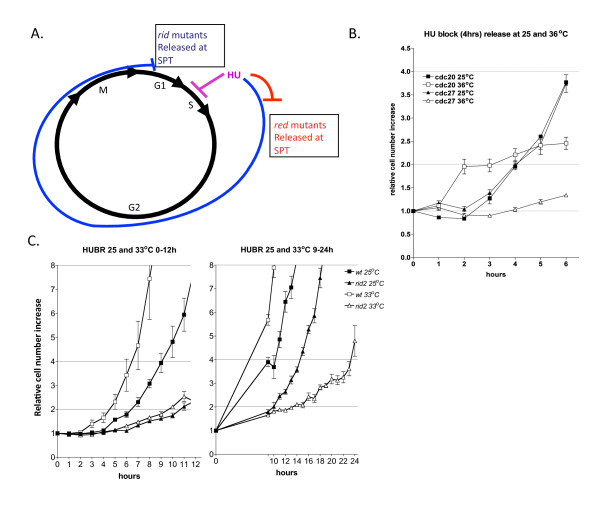
**The *rid2-1 mutant *displays a DNA replication initiation defect**. (A) Experimental Strategy. See Text for a description of the experimental design for the hydroxyurea block and release experiment. (B) Control experiment showing the behavior of two different mutants known to be defective in DNA replication initiation (*cdc20*) and DNA replication elongation (*cdc27*). The cdc20 mutant completes cell division on schedule and doubles following release from HU. As expected for an initiation mutant, no increase in cell number is observed during the second cell cycle period. On the other hand, mutants defective in replication elongation (*cdc27*) show no significant increase in cell number throughout the course of the experiment. (C) Following release from the HU block wildtype cells show normal cell cycle kinetics. The first cell division or population doubling occurs at 4 hrs (33°C) and 6 hrs (25°C) that are longer than normal due to the time needed for recovery from HU. All subsequent cell cycles occur with normal kinetics for the indicated temperatures and are approximately 2 hrs at 33°C and 4 hrs at 25°C. The mutant *rid2-1 *grows slowly and requires at least 10 hours for the first population doubling which is not effected by shifting cells to the semi-permissive temperature of 33°C. However, during the second cell cycle period, *rid2-1 *cells at 33°C have a significant cell cycle delay as compared to cells that remain at 25°C, suggesting that DNA replication initiation is defective in these mutants.

We hypothesized that the *rid *mutant phenotype is specific for mutants defective in DNA replication initiation and is not associated with mutants defective in DNA replication elongation. The fact that our genetic screen yielded two mutants defective in proteins required for DNA replication initiation provides compelling evidence that our hypothesis is correct. However, *rid2-1 *encodes Mcm4, a protein that has also implicated in DNA replication elongation [14]. With the exception of a Mcm-degron mutant that was used to confirm the role of Mcms in replication elongation, all other conditional *mcm *mutants isolated so far have displayed defects only in DNA replication initiation. Nevertheless, we set out to test whether *rid2-1 *has defects in either DNA replication initiation or elongation using a hydroxyurea block and release experimental approach (Figure 5A). Our strategy was to release the *rid2-1 *mutant from a hydroxyurea block at either the semi-permissive temperature (as defined in Materials and Methods) of 25°C or at the higher temperature of 33°C. Considering that HU inhibits DNA replication during the early stages of DNA replication elongation, mutants defective in DNA replication initiation should have no discernable delay in cell cycle progression during the first cell cycle after release from HU. However, a G1/S delay should be observed as cells complete mitosis and enter the subsequent cell cycle. In contrast, mutants defective in DNA replication elongation should display an immediate delay in cell cycle progression upon release from HU. We hypothesized that if shifting the temperature from 25°C to 33°C only affects initiation in the *rid2-1 *mutant, then cell division rates should not change as a function of temperature during the first cell division following release, but as cells enter the subsequent cell cycle, the doubling time should significantly increase at the higher temperature of 33°C.

At 25°C, *rid2-1 *has a cell cycle time nearly double that of wild-type cells that was further increased by treatment with HU. Therefore, in order to examine two complete cell cycles following HU block and release, we needed to extend the time-course following release to 24 hrs. For controls, we examined the cell cycle timing for wild-type cells and for the DNA replication initiation mutant *cdc20 *(encoding DNA polymerase epsilon), and the DNA replication elongation mutant, *cdc27 *(encoding a subunit of DNA polymerase delta) at permissive (wt, *cdc20*, *cdc27*), semi-permissive (wt), or non-permissive temperatures (*cdc20 *and *cdc27*). To demonstrate the expected phenotype for initiation and elongation mutants following HU block and releases, *cdc20 *and *cdc27 *cells were grown at 25°C and treated with HU to arrest cells in early S phase. Cells were then shifted to the non-permissive temperature of 36°C for 1 hr prior to release from HU. Cells defective in DNA polymerase epsilon (*cdc20*) divided once before arresting in the subsequent cell cycle (Figure 5B, open squares). This phenotype is consistent with *cdc20 *cells being defective in DNA replication initiation. In contrast, *cdc27 *cells that are defective in DNA replication elongation fail to divide, and arrest with an elongated cell phenotype consistent with DNA replication being blocked immediately following release from HU (Figure 5B, open triangles). For the experiments involving wt cells and *rid2-1*, cells were first grown overnight at the permissive temperature of 25°C and HU was added for 4 hours to arrest cells in early S phase. Prior to release from HU, half of the cells were shifted to the higher temperature of 33°C while the other half was kept at 25°C. Cells were then filtered and washed with pre-warmed media (25°C or 33°C), and re-suspended in fresh media containing no HU. Cell number was then monitored every hour for approximately 24hrs (or 12 hrs for the wt control). As expected, we found that wild-type cells doubled in approximately 5 hrs (a delay attributable to the HU treatment) and quickly returned to the normal cell cycle time of approximately 2-2.5 hrs during subsequent cell divisions (Figure 5C, open squares). As expected, at 25°C, wt cells have a generation time approximately twice as long as cells grown at the higher temperature of 33°C (Figure 5C, filled squares). Interestingly, for *rid2-1*, no significant cell cycle delay is observed following release from HU at the higher temperature of 33°C (Figure 5C, open triangles) when compared to cells released at the lower temperature of 25°C (Figure 5C, filled triangles), consistent with *rid2-1 *having a negligible effect on DNA replication elongation. However, a strong cell cycle delay is observed for *rid2-1 *cells at 33°C during the subsequent cell cycle. Whereas at 25°C, *rid2-1 *completes the second cell doubling at approximately 15 hrs, at 33°C, cell division does not occur until approximately 24 hrs. Therefore, these results are consistent with *rid2-1 *having a significant defect in DNA replication initiation, whilst having little or no effect on DNA replication elongation, as predicted on the basis of its checkpoint phenotype.

## Discussion

In this report we have shown that mutants defective for DNA replication initiation require Chk1, but not Cds1 to maintain cell viability. We refer to this phenotype as the *rid *phenotype (for replication initiation defective). In contrast, the viability of mutants defective in DNA replication elongation ie. *cdc6*, *cdc27*, is dependent on the checkpoint kinase Cds1. We conducted a genetic screen for mutants that display the *rid *phenotype and isolated two mutants, *rid1-1 *and *rid2-1. *Sequencing confirmed that these mutants contain point mutations in two initiation proteins, Orc1 and Mcm4, respectively. These data are consistent with our screen being highly selective for the isolation of mutants specifically defective in DNA replication initiation. This implies that the cellular response to blocking DNA replication initiation is distinct from the cellular response to inhibiting DNA replication elongation. We have also found that although Cds1 is not essential in mutants defective in DNA replication initiation, the observed dependency on Mrc1 suggests a novel role for this protein in the stabilization of replication forks when initiation is impaired. Mrc1 was originally discovered in both fission yeast and budding yeast as a protein that mediates the activation of Cds1 (Rad53 in *S. cerevisiae*) at stalled replication forks [35-37]. The orthologous protein, Claspin, has a similar role in animal cells [38-41]. Mrc1 has also been shown to be a part of a protein complex that associates with unperturbed replication forks and is believed to have a role in both the stabilization of DNA replication complexes and the coupling of the MCM helicase to DNA polymerase epsilon [5,42-45]. Still other reports suggest that Mrc1 has a role in sister chromatid cohesion [46,47], and telomere capping [48-50]. Based on our analysis of Chk1 activation in *rid *mutants in the absence of Mrc1 we believe that Mrc1 has no role in checkpoint signaling in response to replication initiation defects. However, the dependency of *rid *mutant viability on the presence of Mrc1 strongly argues that the normal replication function(s) of Mrc1 are essential in mutants defective in DNA replication initiation. It is not yet clear whether this reflects its putative role in replication fork stabilization, coupling of the helicase to DNA polymerase or to some other yet unidentified replication function. Moreover, Mrc1 is known to form a complex with at least two additional proteins, Swi1 and Swi3, that could directly interact with the initiation complex and potentially regulate its activity [34,51].

The role of Chk1 in maintaining the viability of *rid *mutants is likely to be its function in delaying the onset of mitosis, however we cannot exclude the possibility that Chk1 might have an additional function in directly stabilizing forks as has been recently observed in *S. cerevisiae *mutants lacking *RAD53 *and *EXO1 *[52].

In *rid2-1*, the mutation in Mcm4 resides in a highly conserved region of the protein called domain 4 of the MCM central homology region. No specific function has been attributed to this region of the protein that lies just outside but adjacent to the Walker A and Walker B motifs that are believed to bind ATP and be important MCM helicase activity [53]. Based on our analysis of the *rid2-1 *mutant, we suggest that this mutation results in a specific defect in DNA replication initiation, but has little effect on the presumed function of Mcms in DNA replication elongation. Consistent with this hypothesis, *rid2-1 *shows normal cell cycle kinetics of DNA replication following release from an early S phase arrest imposed by treatment with hydroxyurea. Therefore, although Mcm proteins are required for both DNA replication initiation and elongation, the data is consistent with *rid2-1 *having defects only in its initiation function. With the exception of the degron mutants of Mcm4, which were specifically designed to test the role of Mcms in replication elongation, our results are similar to those reported for *mcm *mutants in *S. cerevisiae *and *S. pombe *that display no obvious defects in DNA replication elongation. In the case of Orc1, it is generally agreed that Orc1's primary role in DNA replication is to establish pre-RCs at the conclusion of mitosis, and therefore in this case, our screen has led to the identification of a bona-fide initiation protein. This of course does not exclude the possibility that Orc1 might have additional cellular functions outside of DNA replication, as has been recently reported for human Orc1 [54]. The mutation in *rid1-1 *lies in the C-terminal portion of the protein in a region that has yet to be fully characterized.

Understanding the molecular events that lead to the initiation of DNA replication has been a central question for cell biologists for several decades. Recently, several reports have suggested that mutations in the genes that control this step might be associated with genetic instability and cancer. In particular, mutations in MCM4 in mice can lead to increased frequency of sporadic tumors [55]. Moreover, several lines of research have suggested that Mcm proteins might provide important diagnostic markers for early detection of cancer [56-59]. Finally, inhibition of DNA replication might provide an attractive therapeutic strategy for the treatment of cancer, because recent studies have shown that tumor cell lines might be selectively sensitive to inhibition of DNA replication initiation [60-62].

While all previous evidence is consistent with Orc1 having a function in the initiation step of the DNA synthesis, studies on Mcm proteins, including Mcm4, support a dual function during replication, both during the initiation and the elongation stages. Mcm4 was found to interact with ORC components, Cdc18 and Cdc45, as well as with the other MCM complex components (11, 34-37). Mcm4 can be isolated as a heterotrimeric complex with Mcm6 and 7, also known as "the core MCM", that possesses *in vitro *helicase activity and is required for the proper formation of the heterohexameric MCM 2-7 complex (36, 37). Structurally, Mcm4, and all other Mcm subunits, have a central region of homology, known as the MCM domain. In this region, there are the consensus Walker A and B ATPase motifs which are required for nucleotide binding and ATPase activity. Therefore this domain is likely to be responsible for the putative helicase activity of the MCM complex (38-41).

The N-terminal region of Mcm4 presents several putative CDK-phosphorylation sites. Mutational analysis in *Xenopus *and *S. pombe *suggested that phosphorylation of these sites are important for the helicase activity regulation and prevention of re-replication [63-65]. Alignment of several Mcm4 molecules from diverse eukaryote systems showed that the G514E mutation in *rid2-1 *affects a small but very conserved sequence centrally located (in the MCM domain), about 40 amino-acids upstream of the Walker A motif. The G514E mutation lies in the third of five Mcm4 signature motifs that currently has no known function. The high degree of conservation in these signature motifs in many eukaryotes suggests an important yet not determined role for this region of the protein. Further work will be needed to fully understand how the mutation in *rid2-1 *can inhibit initiation of DNA replication. Here we provide a powerful genetic tool for the isolation of mutants defective in DNA replication initiation that should be helpful in identifying new genes required for initiation, as well as hypomorphic alleles of known initiation proteins that can be applied to further analysis of this critical step in cell division.

## Methods

### Strains

All strains used in this study were derived from wildtype *972h- *and *975h+*. All genetic manipulations were performed as previously described [66].

### Nitosoguanidine mutagenesis

Approximately 10^8 ^exponentially growing wildtype or Δ*cds1 *cells were treated with nitrosoguanidine at a final concentration of 0.15 mg/ml. Aliquots of 10^7 ^cells were harvested at different times (0, 5, 10, 15, 20, 30, 45, 60 and 90 minutes) after treatment, were washed three times in Tris-maleate buffer pH6, once in YE and then resuspended in 1 ml YE and incubated at 25°C for four hours to allow recovery. Cells were counted and approximately1000 cells were plated in triplicate for each time-point and incubated at 25°C. After several days, the colonies arising on each plate were counted and a survival curve was plotted. Time 0 was presumed to represent 100% survival. For screening, cells from the 5 minute time-point (representing 50% survival) were plated on rich media plates at a density of approximately 2000 cells per plate and incubated at 25°C until colonies formed. To screen individual colonies for the cell division cycle (*cdc*) phenotype, colonies were replica-plated to YE plates containing phloxine B (a vital stain) and incubated at the elevated temperature of 36°C. The temperature-sensitive (*ts) *colonies (determined by their dark pink staining) were analyzed microscopically and the colonies displaying the *cdc *or elongated cell phenotype were selected as putative *rid *mutants.

### Construction of double mutants

Double mutants were generated using standard procedures [66]. Briefly, cells of different mating types (h^+ ^and h^-^), were mixed on malt extract (ME) plates and incubated at 25°C for 2-3 days. Mature asci were then suspended in 1 ml sterile H_2_O. 5 μl of glusulase were added to the suspension and incubated overnight at 25°C, to allow digestion of the asci walls and to kill all vegetative cells. Spores were then washed twice with sterile H_2_O, plated at a density of 500 spores/plate on rich media (YE) plates and incubated at 25°C to allow spore germination and colony formation. Double mutants were identified after replica plating to confirm the presence of specific markers or phenotypes.

### Viability test (synthetic lethality assessment)

Exponentially growing cultures (OD_595 _= 0.2 - 0.4) for each strain to be analyzed were adjusted to a density of 10^7 ^cells/ml that were then subjected to at least 5 rounds of a 5-fold serial dilution. For each strain, 5 μl of each diluted sample were spotted on YE plates from most concentrated to least concentrated. Plates were incubated at the indicated temperature (ranging between 25 to 36°C) for 4-7 days, or for the amount of time necessary to determine differences between the viability of the strains being tested. For this study, the semi-permissive temperature is defined as the temperature where the single *ts *mutant in the presence or absence of *cds1*^*+ *^is viable, but is sick or inviable in the absence of *chk1*^*+*^.

### Hydroxyurea sensitivity test

Cultures were prepared for each strain as described for the viability experiment. Spots were plated on YE plates containing 0, 2, 5, 7.5, and 10 mM HU, and plates were incubated at temperatures ranging from 25 to 36°C for 4-5 days, and colony formation was assessed.

### Fixing cells for flow cytometry

Cells were harvested by centrifugation at 5000 g for 5 mins, washed once with sterile H_2_O, and re-suspended in 1 ml cold 70% ethanol. For flow cytometry analysis, 0.2 ml of cells in ethanol were centrifuged and re-suspended in re-hydration buffer (50 mM Na citrate containing 0.1 mg/ml RNAase A) and incubated at 37°C for 2 hours. Sytox green (Molecular Probes) was then added to a final concentration of 2 μm prior to analysis.

### Fission yeast transformation

Exponentially growing cells (OD_595 _= 0.2-0.5) were harvested, washed once with sterile water, re-suspended at a density of 10^9 ^cells/ml in 0.1 M lithium acetate (pH 4.9) and dispensed in 100 μl aliquots into Eppendorf tubes. Cells were incubated at 25°C for 60 - 120 minutes. 1 μg of plasmid DNA in 15 μl TE (pH 7.5) was added to each aliquot and mixed, followed by the addition of 290 μl of 50% (w/v) PEG 4000, mixing and incubation at 25°C for 60 minutes. Cells were heat shocked at 43°C for 15 minutes. Tubes were cooled to room temperature for 10 minutes, and then cells were harvested by centrifugation, and re-suspended in 1 ml of 1/2 YE broth. The suspension was transferred to a 50 ml flask and diluted with 9 ml of 1/2 YE, followed by recovery at 25°C for 60 minutes. Cells were then concentrated in 1 ml of ½ YE and 100 ul aliquots were plated on selective agar plates.

### Cloning of *rid1-1 *and *rid2-1 *mutated genes

*Genomic DNA extraction from rid1-1 and rid2-1 strains: rid1-1 *and *rid2-1 *liquid cultures were grown to stationary phase. Cells were harvested, re-suspended in 50 mM citrate/phosphate pH 5.6 40 mM EDTA pH 8.0 1.2 M sorbitol, followed by the addition of Zymolase and incubated at 37°C for 30-60 minutes. The cells were then pelleted briefly and resuspended in TE buffer 1% SDS and incubated at 65°C for 1 hr. 5 M potassium acetate was added, and the tube was kept on ice for 5 minutes. The resulting suspension was centrifuged at 12000 rpm for 15 minutes at 4°C, the supernatant was collected and the DNA was precipitated by addition of cold isopropanol and incubation at -20°C for 10 minutes. The tube was then centrifuged for 15 minutes at 4°C. The supernatant was discarded and the pellet was washed with 70% ETOH. The pellet was resuspended in TE buffer containing 50 μg/ml RNAse A and incubated at 65°C for 10 minutes. Two rounds of phenol/chloroform extraction were performed, followed by 3 M sodium acetate and cold 100% ethanol precipitation of DNA on ice for 10 minutes. The DNA was pelleted by centrifugation at 10000 rpm for 10 minutes, washed with 70% ETOH and dried at 37°C. The last step was the resuspension of genomic DNA in TE buffer containing RNAse A and incubating for 5 minutes at 37°C.

*Primer design: *The primers used for PCR amplification of the entire *cdc21/mcm4 *and *cdc30/orp1 *open reading frames were:

*mcm4 Xho*I-F: 5' CCGCTCGAGCGGTCTTTCTACACCCACCACGAG 3'

*mcm4 Bam*HI-R: 5' CGCGGATCCGCGGAATAATACCAGCTTATTCGC 3'

*orp1Bam*HI-F: 5' CGCGGATCCATGCCTAGAAGAAAGTCATTG 3'

orp1*Xma*I-R: 5' TCCCCCCGGGTTATGCTATCCCAGCAAGTTCC 3'

*Cloning rid1-1 and rid2-1 in pCR Blunt vector: *PCR amplification of *rid1-1 *(mutated *cdc30/orp1 *gene) and *rid2-1 *(mutated *cdc21/mcm4 *gene) was completed using the above primers and genomic DNA extracted using *rid1-1 *and *rid2-1 *strains as the template DNA. After checking the proper band size on a 0.8% agarose gel, the ligation was performed directly from the PCR product into a pCR-Blunt vector (Invitrogen Topo-Blunt kit).

### Sequencing the *rid1 *(*cdc30/orp1*) and *rid2 *(*cdc21/mcm4*) gene

*Primer design: *Additional forward and reverse primers were designed for *cdc30 *and *cdc21*, spanning the entire gene lengths, spaced at every 600bp:

*orp1*-501-F: ACGAAAAGATTTGTTTCCTT

*orp1*-541-R: TTCACTTTCAAGTGTACACC

*orp1*-1101-F: TGGTACGCCGGGAACAGGAA

*orp1*-1141-R: TCCTGAAGATTCCAAATTAC

*orp1*-1701-F: AGAGCTTGCTGAAAACAAAA

*orp1*-1741-R: GAAATTGCTTGATGAATTAA

*mcm4*-501-F: AGAAAGTATCGCTTCCTTTC

*mcm4*-541-R: TCAGGTCGATACTTTTTCTT

*mcm4*-1101-F: ATTCGCCGATAAGCAAGTTA

*mcm4*-1141-R: TGGCCATCCGGTACCACGTC

*mcm4*-1701-F: CACTAGTGGCAAGGGTTCAT

*mcm4*-1741-R: TCTTGGTCACGAGTAATATA

*mcm4*-2301-F: AATTACTGCTACAACAAGAC

*mcm4*-2341-R: TTCGCATGGGCCTCAGATAG

Other primers used were the M13 forward and reverse primers existing in the pCR-Blunt vector, flanking the cloning sites.

*Sequencing and data analysis*: Cloned DNA from each mutant were sequenced and aligned with the wildtype *cdc30*^*+ *^and *cdc21*^*+ *^sequences using the Clone Manager Suite 7 software to identify the site of the mutations in *rid1-1 *and *rid2-1*. The same software was used to translate and compare the amino acid sequences of wildtype and mutant Orc1 and Cdc21 proteins.

### HU block-release

Exponentially growing cultures were treated with 12 mM HU for 4 hours, followed by HU wash by filtration under vacuum on Millipore membranes, with media warmed to the temperature to be used for shift. For each experiment, after wash, cells were shifted to the indicated temperatures, and aliquots were collected every 30 minutes for cell counting. The length of temperature exposure depended on the time needed for *wt *(used as control for each experiment and every temperature tested) and mutant strains to undergo two complete rounds of cell division. Samples were fixed in formal saline and then the number of cells in a fixed volume was counted using Sysmex K-1000, in triplicate, for each sample.

## Competing interests

The authors declare that they have no competing interests.

## Authors' contributions

AML completed the *rid *mutant screen, cloned and sequenced the *rid *mutants, and did all physiology experiments. LY tested checkpoint sensitivity of the rid strains and analyzed Chk1 activation in *rid *mutants. LY also assisted in the genetic screens. GD was responsible for designing the screen, and overseeing all experiments. GD and AML were responsible for writing the manuscript and preparing all figures. LY provided the data for Figure 4. All authors read and approved the final manuscript.

## Supplementary Material

Additional file 1 ***S. pombe *mutants tested for *rid *phenotype**. Click here for file

## References

[B1] Diffley JF Regulation of early events in chromosome replication Curr Biol 2004 14 18 R778 86 10.1016/j.cub.2004.09.019 15380092

[B2] Cvetic CA Walter JC Getting a grip on licensing: mechanism of stable Mcm2-7 loading onto replication origins Mol Cell 2006 21 2 143 4 10.1016/j.molcel.2006.01.003 16427002

[B3] Wohlschlegel JA Inhibition of eukaryotic DNA replication by geminin binding to Cdt1 Science 2000 290 5500 2309 12 10.1126/science.290.5500.2309 11125146

[B4] Randell JC Sequential ATP hydrolysis by Cdc6 and ORC directs loading of the Mcm2-7 helicase Mol Cell 2006 21 1 29 39 10.1016/j.molcel.2005.11.023 16387651

[B5] Hodgson B Calzada A Labib K Mrc1 and Tof1 regulate DNA replication forks in different ways during normal S phase Mol Biol Cell 2007 18 10 3894 902 10.1091/mbc.E07-05-0500 17652453 PMC1995724

[B6] Gambus A GINS maintains association of Cdc45 with MCM in replisome progression complexes at eukaryotic DNA replication forks Nat Cell Biol 2006 8 4 358 66 10.1038/ncb1382 16531994

[B7] Fien K Primer utilization by DNA polymerase alpha-primase is influenced by its interaction with Mcm10p J Biol Chem 2004 279 16 16144 53 10.1074/jbc.M400142200 14766746

[B8] Ricke RM Bielinsky AK Mcm10 regulates the stability and chromatin association of DNA polymerase-alpha Mol Cell 2004 16 2 173 85 10.1016/j.molcel.2004.09.017 15494305

[B9] Uchiyama M Arai K Masai H Sna41goa1, a novel mutation causing G1/S arrest in fission yeast, is defective in a CDC45 homolog and interacts genetically with polalpha Mol Genet Genomics 2001 265 6 1039 49 10.1007/s004380100499 11523776

[B10] Uchiyama M Essential role of Sna41/Cdc45 in loading of DNA polymerase alpha onto minichromosome maintenance proteins in fission yeast J Biol Chem 2001 276 28 26189 96 10.1074/jbc.M100007200 11344166

[B11] Pacek M Walter JC A requirement for MCM7 and Cdc45 in chromosome unwinding during eukaryotic DNA replication EMBO J 2004 23 18 3667 76 10.1038/sj.emboj.7600369 15329670 PMC517609

[B12] Zegerman P Diffley JF Phosphorylation of Sld2 and Sld3 by cyclin-dependent kinases promotes DNA replication in budding yeast Nature 2007 445 7125 281 5 10.1038/nature05432 17167417

[B13] Tanaka S CDK-dependent phosphorylation of Sld2 and Sld3 initiates DNA replication in budding yeast Nature 2007 445 7125 328 32 10.1038/nature05465 17167415

[B14] Labib K Diffley JF Is the MCM2-7 complex the eukaryotic DNA replication fork helicase? Curr Opin Genet Dev 2001 11 1 64 70 10.1016/S0959-437X(00)00158-1 11163153

[B15] Shechter D Ying CY Gautier J DNA unwinding is an Mcm complex-dependent and ATP hydrolysis-dependent process J Biol Chem 2004 279 44 45586 93 10.1074/jbc.M407772200 15326181

[B16] Boddy MN Russell P DNA replication checkpoint Curr Biol 2001 11 23 R953 6 10.1016/S0960-9822(01)00572-3 11728320

[B17] Al-Khodairy F Carr AM Mutants defining the G2 checkpoint pathway in S. pombe EMBO J 1992 11 4 1343 1350 1563350 10.1002/j.1460-2075.1992.tb05179.x PMC556583

[B18] Zeng Y Replication checkpoint requires phosphorylation of the phosphatase Cdc25 by Cds1 or Chk1 Nature 1998 395 6701 507 10 10.1038/26766 9774107

[B19] Brondello JM Basis for the checkpoint signal specificity that regulates Chk1 and Cds1 protein kinases Mol Cell Biol 1999 19 6 4262 4269 10330167 10.1128/mcb.19.6.4262 PMC104386

[B20] Boddy MN Replication checkpoint kinase Cds1 regulates recombinational repair protein Rad60 Mol Cell Biol 2003 23 16 5939 46 10.1128/MCB.23.16.5939-5946.2003 12897162 PMC166335

[B21] Rhind N Russell P Chk1 and Cds1: linchpins of the DNA damage and replication checkpoint pathways J Cell Sci 2000 113 Pt 22 3889 96 11058076 10.1242/jcs.113.22.3889 PMC2863124

[B22] 280Boddy MN Replication checkpoint enforced by kinases Cds1 and Chk1 Science 1998 280 5365 909 912 10.1126/science.280.5365.909 9572736

[B23] Mochida S Regulation of checkpoint kinases through dynamic interaction with Crb2 EMBO J 2004 23 2 418 28 10.1038/sj.emboj.7600018 14739927 PMC1271744

[B24] Walworth NC DNA damage: Chk1 and Cdc25, more than meets the eye Curr Opin Genet Dev 2001 11 1 78 82 10.1016/S0959-437X(00)00160-X 11163155

[B25] Feng W D'Urso G Schizosaccharomyces pombe cells lacking the amino-terminal catalytic domains of DNA polymerase epsilon are viable but require the DNA damage checkpoint control Mol Cell Biol 2001 21 14 4495 504 10.1128/MCB.21.14.4495-4504.2001 11416129 PMC87109

[B26] Yin L Locovei AM D'Urso G Activation of the DNA damage checkpoint in mutants defective in DNA replication initiation Mol Biol Cell 2008 19 10 4374 82 10.1091/mbc.E08-01-0020 18667534 PMC2555949

[B27] Hirano T Isolation and characterization of Schizosaccharomyces pombe cutmutants that block nuclear division but not cytokinesis EMBO J 1986 5 11 2973 2979 16453724 10.1002/j.1460-2075.1986.tb04594.x PMC1167249

[B28] Fletcher RJ The structure and function of MCM from archaeal M. Thermoautotrophicum Nat Struct Biol 2003 10 3 160 7 10.1038/nsb893 12548282

[B29] Fletcher RJ Identification of amino acids important for the biochemical activity of Methanothermobacter thermautotrophicus MCM Biochemistry 2008 47 38 9981 6 10.1021/bi800032t 18754676 PMC2639774

[B30] Shikata M Ishikawa F Kanoh J Tel2 is required for activation of the Mrc1-mediated replication checkpoint J Biol Chem 2007 282 8 5346 55 10.1074/jbc.M607432200 17189249

[B31] Shimmoto M Interactions between Swi1-Swi3, Mrc1 and S phase kinase, Hsk1 may regulate cellular responses to stalled replication forks in fission yeast Genes Cells 2009 14 6 669 82 10.1111/j.1365-2443.2009.01300.x 19422421 PMC2837079

[B32] Krings G Bastia D swi1- and swi3-dependent and independent replication fork arrest at the ribosomal DNA of Schizosaccharomyces pombe Proc Natl Acad Sci USA 2004 101 39 14085 90 10.1073/pnas.0406037101 15371597 PMC521093

[B33] Noguchi C Noguchi E Sap1 Promotes the Association of the Replication Fork Protection Complex With Chromatin and Is Involved in the Replication Checkpoint in Schizosaccharomyces pombe Genetics 2007 175 2 553 66 10.1534/genetics.106.065334 17151242 PMC1800610

[B34] Dalgaard JZ Klar AJ swi1 and swi3 perform imprinting, pausing, and termination of DNA replication in S. pombe Cell 2000 102 6 745 51 10.1016/S0092-8674(00)00063-5 11030618

[B35] Alcasabas AA Mrc1 transduces signals of DNA replication stress to activate Rad53 Nature Cell Biology 2001 3 11 958 65 10.1038/ncb1101-958 11715016

[B36] Osborn AJ Elledge SJ Mrc1 is a replication fork component whose phosphorylation in response to DNA replication stress activates Rad53 Genes Dev 2003 17 14 1755 67 10.1101/gad.1098303 12865299 PMC196183

[B37] Tanaka K Russell P Mrc1 channels the DNA replication arrest signal to checkpoint kinase Cds1 Nat Cell Biol 2001 3 11 966 72 10.1038/ncb1101-966 11715017

[B38] Kumagai A Dunphy WG Repeated phosphopeptide motifs in Claspin mediate the regulated binding of Chk1 Nat Cell Biol 2003 5 2 161 5 10.1038/ncb921 12545175

[B39] Kumagai A Dunphy WG Claspin, a novel protein required for the activation of Chk1 during a DNA replication checkpoint response in Xenopus egg extracts Mol Cell 2000 6 4 839 49 10.1016/S1097-2765(05)00092-4 11090622

[B40] Kumagai A Kim SM Dunphy WG Claspin and the activated form of ATR-ATRIP collaborate in the activation of Chk1 J Biol Chem 2004 279 48 49599 608 10.1074/jbc.M408353200 15371427

[B41] Lee J Roles of replication fork-interacting and Chk1-activating domains from Claspin in a DNA replication checkpoint response Mol Biol Cell 2005 16 11 5269 82 10.1091/mbc.E05-07-0671 16148040 PMC1266425

[B42] Labib K Making connections at DNA replication forks: Mrc1 takes the lead Mol Cell 2008 32 2 166 8 10.1016/j.molcel.2008.10.005 18951084

[B43] Tourriere H Mrc1 and Tof1 promote replication fork progression and recovery independently of Rad53 Mol Cell 2005 19 5 699 706 10.1016/j.molcel.2005.07.028 16137625

[B44] Bando M Csm3, Tof1, and Mrc1 form a heterotrimeric mediator complex that associates with DNA replication forks J Biol Chem 2009 284 49 34355 65 10.1074/jbc.M109.065730 19819872 PMC2797203

[B45] Lou H Mrc1 and DNA polymerase epsilon function together in linking DNA replication and the S phase checkpoint Mol Cell 2008 32 1 106 17 10.1016/j.molcel.2008.08.020 18851837 PMC2699584

[B46] Xu H Boone C Brown GW Genetic dissection of parallel sister-chromatid cohesion pathways Genetics 2007 176 3 1417 29 10.1534/genetics.107.072876 17483413 PMC1931553

[B47] Xu H Boone C Klein HL Mrc1 is required for sister chromatid cohesion to aid in recombination repair of spontaneous damage Mol Cell Biol 2004 24 16 7082 90 10.1128/MCB.24.16.7082-7090.2004 15282308 PMC479732

[B48] Tsolou A Lydall D Mrc1 protects uncapped budding yeast telomeres from exonuclease EXO1 DNA Repair (Amst) 2007 6 11 1607 17 10.1016/j.dnarep.2007.05.010 17618841 PMC2077361

[B49] Grandin N Bailly A Charbonneau M Activation of Mrc1, a mediator of the replication checkpoint, by telomere erosion Biol Cell 2005 97 10 799 814 10.1042/BC20040526 15760303

[B50] Grandin N Charbonneau M Mrc1, a non-essential DNA replication protein, is required for telomere end protection following loss of capping by Cdc13, Yku or telomerase Mol Genet Genomics 2007 277 6 685 99 10.1007/s00438-007-0218-0 17323081

[B51] Noguchi E Swi1 and Swi3 are components of a replication fork protection complex in fission yeast Mol Cell Biol 2004 24 19 8342 55 10.1128/MCB.24.19.8342-8355.2004 15367656 PMC516732

[B52] Segurado M Diffley JF Separate roles for the DNA damage checkpoint protein kinases in stabilizing DNA replication forks Genes Dev 2008 22 13 1816 27 10.1101/gad.477208 18593882 PMC2492668

[B53] Forsburg SL Mutational analysis of Cdc19p, a Schizosaccharomyces pombe MCM protein Genetics 1997 147 3 1025 41 9383050 10.1093/genetics/147.3.1025 PMC1208231

[B54] Prasanth SG Prasanth KV Stillman B Orc6 involved in DNA replication, chromosome segregation, and cytokinesis Science 2002 297 5583 1026 31 10.1126/science.1072802 12169736

[B55] Shima N A viable allele of Mcm4 causes chromosome instability and mammary adenocarcinomas in mice Nat Genet 2007 39 1 93 8 10.1038/ng1936 17143284

[B56] Scarpini C Improved screening for anal neoplasia by immunocytochemical detection of minichromosome maintenance proteins Cancer Epidemiol Biomarkers Prev 2008 17 10 2855 64 10.1158/1055-9965.EPI-08-0288 18843031

[B57] Mukherjee G MCM immunocytochemistry as a first line cervical screening test in developing countries: a prospective cohort study in a regional cancer centre in India Br J Cancer 2007 96 7 1107 11 10.1038/sj.bjc.6603679 17342084 PMC2360130

[B58] Scott IS A minimally invasive immunocytochemical approach to early detection of oral squamous cell carcinoma and dysplasia Br J Cancer 2006 94 8 1170 5 10.1038/sj.bjc.6603066 16622441 PMC2361243

[B59] Gonzalez MA Minichromosome maintenance protein 2 is a strong independent prognostic marker in breast cancer J Clin Oncol 2003 21 23 4306 13 10.1200/JCO.2003.04.121 14645419

[B60] Swords R Cdc7 kinase - A new target for drug development Eur J Cancer 2009 10.1016/j.ejca.2009.09.020 19815406

[B61] Montagnoli A A Cdc7 kinase inhibitor restricts initiation of DNA replication and has antitumor activity Nat Chem Biol 2008 4 6 357 65 10.1038/nchembio.90 18469809

[B62] Montagnoli A Cdc7 inhibition reveals a p53-dependent replication checkpoint that is defective in cancer cells Cancer Res 2004 64 19 7110 6 10.1158/0008-5472.CAN-04-1547 15466207

[B63] Pereverzeva I Distinct phosphoisoforms of the Xenopus Mcm4 protein regulate the function of the Mcm complex Mol Cell Biol 2000 20 10 3667 76 10.1128/MCB.20.10.3667-3676.2000 10779356 PMC85659

[B64] Ishimi Y Levels of MCM4 phosphorylation and DNA synthesis in DNA replication block checkpoint control J Struct Biol 2004 146 1-2 234 41 10.1016/j.jsb.2003.11.027 15037254

[B65] Komamura-Kohno Y Site-specific phosphorylation of MCM4 during the cell cycle in mammalian cells FEBS J 2006 273 6 1224 39 10.1111/j.1742-4658.2006.05146.x 16519687

[B66] Moreno S Klar A Nurse P Molecular genetic analysis of fission yeast Schizosaccharomyces pombe Method. Enzymol 1991 194 795 795 23 full_text 10.1016/0076-6879(91)94059-l 2005825

