# Supplementary material for: A genetic screen for replication initiation defective (rid) mutants in Schizosaccharomyces pombe
Source: Cell Div. 2010 Aug 27;5:20. doi: 10.1186/1747-1028-5-20 (PMC2940899; doi:10.1186/1747-1028-5-20)
Supplement: Additional file 1 — S. pombe mutants tested for rid phenotype. [file 1747-1028-5-20-S1.PDF]

Table 1. *S. pombe* mutants tested for *rid* phenotype. (+) viable at semi-permissive temperature, (-), inviable at semi-permissive temperature (+/-), moderate loss of viability (-*), inviable, confirmed by tetrad analysis.

S.pombe strain	Gene product	*Δcds1*	*Δchk1*
Wild type		+	+
Initiation Mutants			
cdc20 ΔNterm	DNA pol. ε large subunit	+	–
cdc20-P7	DNA pol. ε large subunit	+	–
cdc20-M10	DNA pol. ε large subunit	+	–
cdc30-2H4	ORC subunit 1	+	–
orp2-2	ORC subunit 2	+	–
orp2-7	ORC subunit 2	+	–
orp5-H19	ORC subunit 5	+	–
cdc18-K46	S phase initiator	+	–
cdc21-M68	Mcm4 (MCM4 class)	+	–
mis5-268	Mcm6 (MCM6 class)	+	–*
cdc23-M36	MCM10 homologue	+	–
sna41-912	Cdc45 homologue	+	–
Elongation Mutants			
cdc6-23	DNA pol. δ, large (catalytic) subunit	–	+/–
polδ ts1	DNA pol. δ, large (catalytic) subunit	–	+/–
cdc27-M57	DNA pol. δ associated factor	–	–
cdc22-M45	ribonucleotide reductase, large subunit	–*	+/–
cdc24-81	removal of RNA primer (no homology with *S. cerevisiae*.)	–	–
